# Risk analysis of African swine fever in Poland based on spatio-temporal pattern and Latin hypercube sampling, 2014–2017

**DOI:** 10.1186/s12917-019-1903-z

**Published:** 2019-05-22

**Authors:** Yi Lu, Xiaojun Deng, Jiahui Chen, Jianying Wang, Qin Chen, Bing Niu

**Affiliations:** 1 School of Life Sciences, Shanghai University, Shanghai, 200444 China; 2 Technology Center for Animal, Plant and Food Inspection and Quarantine, Shanghai Entry Exit Inspect and Quarantine Bur, Shanghai, 200135 China

**Keywords:** African swine fever, Risk analysis, Standard deviation ellipse, Space-time scan statistical, Latin hypercube sampling

## Abstract

**Background:**

African swine fever (ASF) is a devastating infectious disease of pigs. ASF poses a potential threat to the world pig industry, due to the lack of vaccines and treatments. In this study, the Geographic Information System (GIS) spatial analysis was applied to analyze the distribution, dispersion of the epidemic and clustering of ASF in Poland.

**Results:**

The results show that the center of the epidemic moved gradually towards the southwest, and the distribution of the epidemic changed from south-north to east-west. Through space-time scan statistical analysis, the 3 clusters major of wild boar cases involve longer time spans and larger radii, while the other five with higher relative risks involved in domestic pigs. And then, a quantitative model was constructed to analyse the risk of releasing African swine fever virus (ASFV) from Poland by the legal export of pork and pork products. The Latin hypercube sampling results show that the probability is relatively low (the average value is 4.577 × 10^− 7^).

**Conclusions:**

All the identification of the spatio-temporal patterns of the epidemic and the risk analysis model would give a further understanding of the dynamics of disease transmission and help to design corresponding measures to minimize the catastrophic consequences of potential ASFV introduction.

## Background

African swine fever (ASF) is the most serious epidemic disease in the pig industry, which is caused by the African swine fever virus (ASFV) [1]. Suids are the only susceptible animals to ASFV. The morbidity and mortality caused by infection with a virulent strain of the virus can be as high as 100% and it is listed in the World Organisation for Animal Health (OIE) Terrestrial Animal Health Code and must be reported to the OIE Terrestrial Animal Health Code [2]. The clinical symptoms can be divided into different categories according to severity, namely most acute, acute, subacute, chronic and recessive. The main clinical manifestations are hemorrhage of skin, mucous membrane, internal organs and respiratory disorder, and these symptoms are very similar to those reported for classical swine fever, porcine erysipelas, coumarin poisoning and hemorrhagic purpura. It is very easy to misdiagnose based only on clinical symptoms and therefore laboratory confirmation is needed for accurate diagnosis.

At the same time, ASF is a disease with obvious regional epidemic. The first strain of ASFV was found in Kenya in 1921 [3]. The outbreaks were initially concentrated in eastern Africa, and then the outbreak continued to erupt in the southern African countries. Up to now ASF is still endemic in southeastern Africa [4]. In 1957, it first appeared in Portugal through the swill in aircrafts, and the virus has spread gradually into Western Europe, in countries such as Spain and Italy. In 2007, ASF was introduced into Georgia. Subsequently, an ASF epidemic occurred in neighboring Armenia, Russia and Azerbaijan, and thus formed a new prevalence area for its establishment [5].

**Fig. 1:**
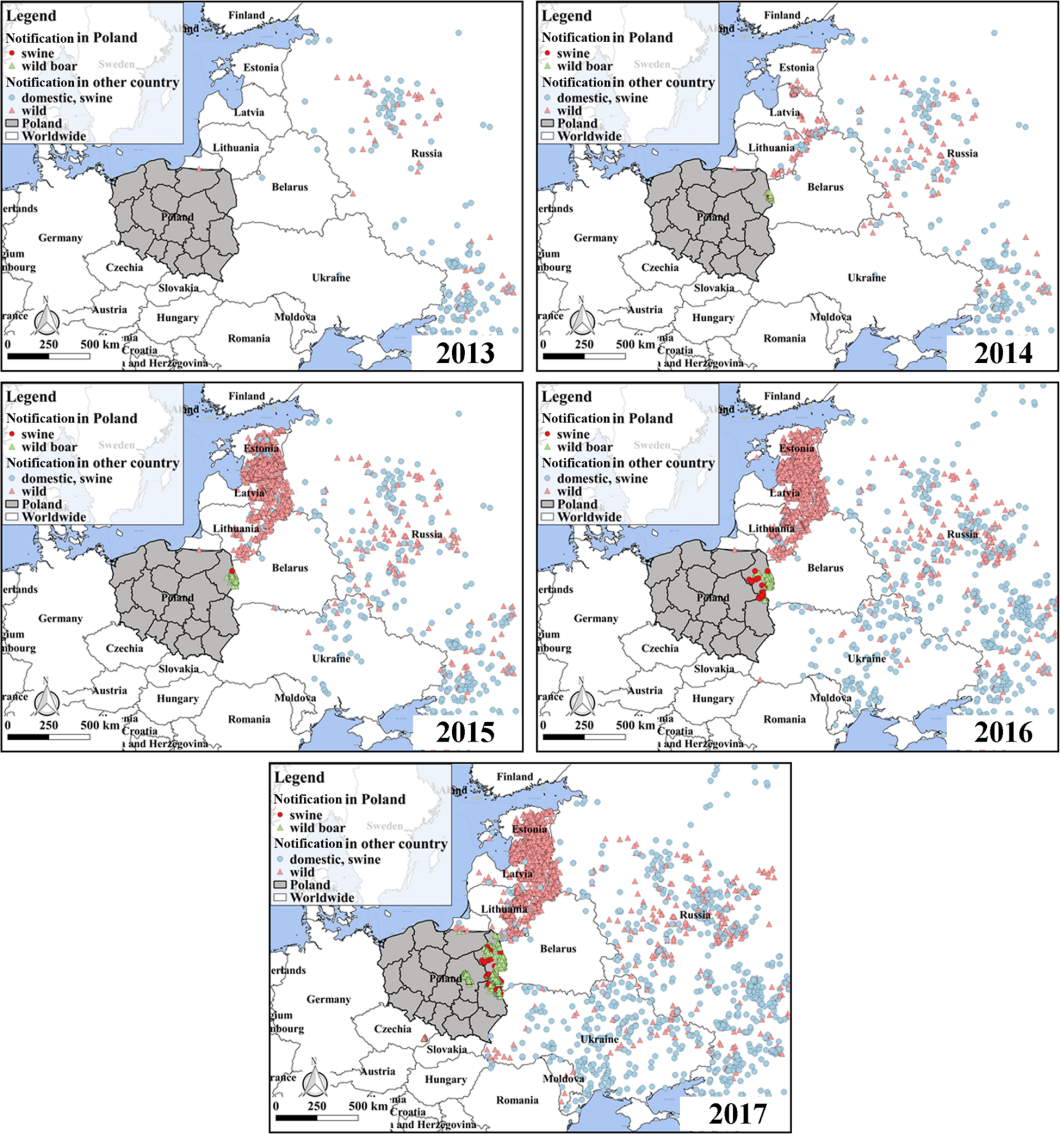
The outbreak of ASF in Poland and its neighboring countries, 2013–2017. The maps were generated with QGIS Version 2.18 (https://www.qgis.org/en/site/index.html) and the base map was Bing Maps from QuickMapServices

Since Russia officially notified the authorities about the ASF epidemic in June 2007, the neighboring countries have taken corresponding measures such as banning the trading of live pigs and pig products, to prevent the introduction of ASFV into their territories for the first time, but the ASF is still spreading rapidly in Europe through other means. By the end of 2013, the outbreaks of ASF in Europe were concentrated in Russia with a few cases occurring in Ukraine and Belarus. In 2014, the European Union (EU) countries such as Lithuania, Poland and Estonia reported the presence of ASF. During the 2015–2016, the epidemic continued to spread in Eastern Europe, especially in Estonia, Lithuania and Latvia. In 2017, the Czech Republic and Romania reported the first cases of ASF in wild boars and domestic pigs respectively [6]. By the end of 2017, 11 European countries had had ASF epidemics in succession, and the ASF epidemic continues to show a tendency to move further westward (Fig. 1). In February 2014, the body of ASF infected wild boar was first discovered in the Podlaskie Province, about 900 m from the Belarusian border, and the ASF began to spread in Poland [7, 8]. By the year 2017, four provinces in Poland had reported ASF outbreaks. The total outbreaks are more than 900 from 2014 to 2017.

Since there is no effective vaccine and treatment method for ASF, the prevention and control of this disease mainly relies on laboratory methods for its rapid and accurate detection methods and the subsequent strict killing and hygiene regimens employed [9, 10]. Several studies have shown that domestic pigs are very sensitive to the direct and indirect infection by the ASFV strains currently prevalent in Eastern Europe and this has led to the development of acute forms of the disease [11–13]. Meanwhile, pork and pork products (especially domestic pigs) are important agricultural products. The existence of ASF poses a severe impact on national and international policies and trade restrictions, and can have a devastating impact on animal health and the world economy [14]. The prevalence of ASF in the Caucasus has threatened Europe, Central Asia and even China the country with the largest number of pigs on hand and total production in the world. On August, 2018, an outbreak of African swine fever in pigs was reported in China Liaoning province. The phylogenetic analysis showed that the p72 genotype II of the causative strain had extremely high homology with those of other genotype II strains, and there is just a 10-bp additional fragment inserted into Georgia 2007 strain, suggesting the origin of this strain from a homogenotypic strain [15].

In 2012, six pork production and processing companies in Poland were allowed to register in China, and Polish pork products entered the official Chinese market. In 2013, China imported 50,000 tons of Polish pork, which is the largest pork export market in Poland. However, in response to this sudden epidemic in Poland, and the source of the infection is not yet known, the Ministry of Agriculture and General Administration of Quality Supervision, Inspection and Quarantine issued an announcement on February 24, 2014 to prohibit the import of all Polish pork and pork products [16]. The government issued an administrative order to control the source of infectious diseases, eliminating all hidden dangers that ASFV might introduce.

Since the current epidemic in Poland is only concentrated in the eastern region of the country, and most of them are wild boar epidemics, the Polish government has taken emergency measures such as delineating buffer zones to actively prevent the spread of the epidemic. In response to the related regulations of the WTO/SPS [17], measures were taken to minimize the negative impact on trade on the basis of ensuring the life and health of human beings, animals or plants.

In this paper, in order to investigate the epidemic characteristics and the directional trend and identify the significant presence of spatial-temporal clustering of the ASF, GIS-based standard deviational ellipse analyses and SaTScan-based retrospective space-time permutation scan statistics were conducted. And then a quantitative risk analysis model was built to further analyze the probability of Polish pork and pork products carrying ASFV exported through legal channels. The results of these analyses would help to identify the potential risks of ASFV exports from Poland and provide recommendations for preventive measures to avoid the introduction and spreading of ASF.

## Methods

### Distribution and clustering of the epidemic situation

The surveillance data on ASF from February 2014 to December 2017 in Poland were collated from the EMPRES Global Animal Disease Information System (EMPRES-i) [18] of the United Nations Food and Agriculture Organization (FAO), since the actual longitude and latitude of the outbreak can be obtained. QGIS 2.18 was used to perform the directional trend analysis of epidemic outbreaks and SaTScan version 9.5 was conducted for space-time permutation scan statistics.

### Standard deviation ellipse analysis

Standard Deviation Ellipse (SDE) Analysis is also known as directional distribution analysis. It can measure the spatial dispersion of a set of geographical events very well and it provides useful information on the directional bias of their locations or expansions [19, 20]. The spatial characteristics of ASF such as central trends, dispersion and orientation trends can be analyzed by creating ellipses. The ellipse shows the spatial expansion of a set of point locations and helps us to determine whether the distribution of these points is elongated so to extend in a particular direction. The ratio of the long axis to the short axis reflects the apparent degree of clustering or dispersion of the ASF epidemic. A ratio of greater than 1 indicates apparent orientational effects of the epidemic distribution, and when the ratio equal to 1, this means there is no obvious directional characteristics [21, 22]. In this study, the size of the ellipse was set to cover 68% of the flash point data to study the size of the ASF epidemic area and the directional trend that the ASF epidemic presented during 2014–2017.

### Space-time scan statistics

In this paper, a space–time permutation scan statistical model was applied to study the relationship of the ASF epidemic on time and space in Poland. The space–time permutation scan statistic only needs a precise spatial location and a time period, but does not require any relevant risk population data. Due to the lack of population data of risky animals, the space–time permutation scan statistic of SaTScan 9.4.2 was used to analyze the spatial-temporal clustering of the epidemic.

The scan window in the space–time permutation scan statistical model is a cylinder whose size at the bottom circle represents the area of the space and the height represents the length of time [23]. In this study, the window was moved in time and space to cover each possible geographic location and time interval, and model parameters for maximum space and time window size were set so that the cluster area could include up to 50% ASF cases. In the analysis process, the log likelihood ratio statistics (LLR) [24] was used to evaluate whether the cylinder contains a cluster area, and the Monte Carlo simulation was used to evaluate the significance of the detected cluster *p*-value [25]. To ensure sufficient accuracy, the number of Monte Carlo simulations was set to 999, and the potential disease spread was reflected in days. All the clustering results in the paper were with *p*-values of less than 0.01. Finally, the relative risk value (RR) is calculated by using formula (1), which is used to represent the risk of the cluster relative to the diseased area outside the cluster.

1 $$ \mathrm{RR}=\frac{C_C/E\left({C}_C\right)}{\left({C}_T-{C}_C\right)/\left(E\left({C}_T\right)-E\left({C}_C\right)\right)}=\frac{C_C/E\left({C}_C\right)}{\left({C}_T-{C}_C\right)/\left({C}_T-E\left({C}_C\right)\right)} $$

where *C*_*C*_ is the number of observed cases within the cluster and *C*_*T*_ is the total number of cases. *E*(*C*_*C*_) is the number of excepted cases within the cluster and *E*(*C*_*T*_) is the total excepted cases in the data set. Note that since the analysis is conditioned on the total number of cases observed, *E*(*C*_*T*_) = *C*_*T*_.

### Quantitative risk analysis model

**Fig. 2:**
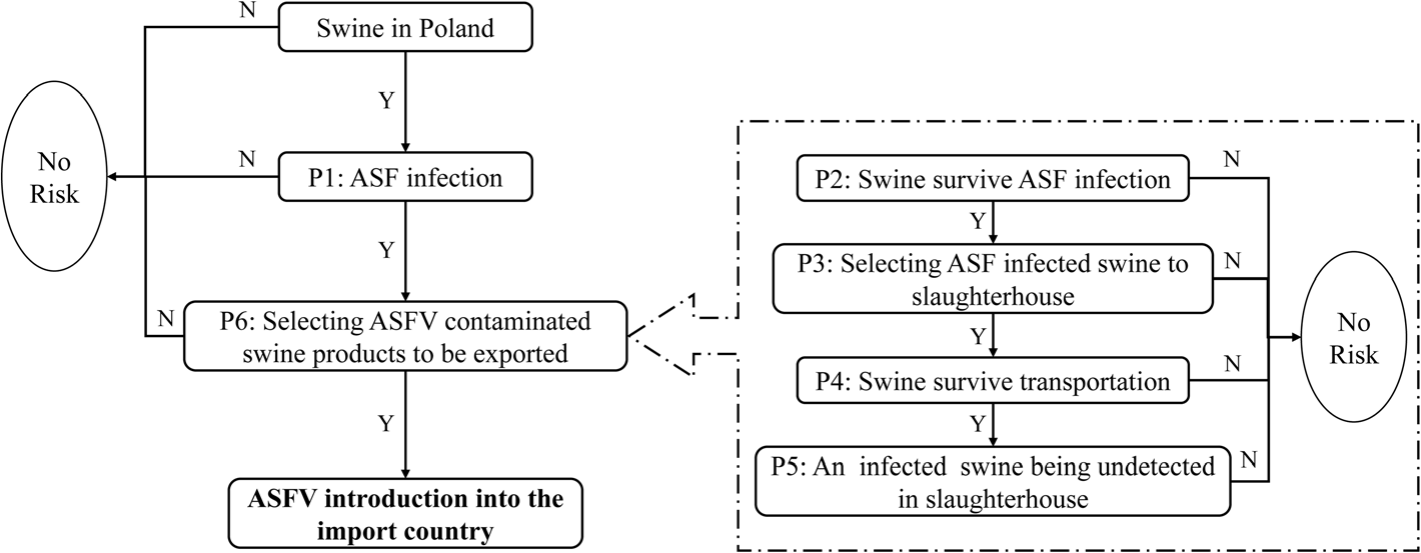
Risk assessment of releasing ASFV by legal export of pork and pork products from Poland

According to the relevant guidelines of OIE [26], the risk assessment process is divided into three steps: entry (or release) assessment, exposure assessment and consequence assessment. Due to data limitations and not limited to incoming countries, this article only evaluates release risks. Data on trade such as import and export of Polish pork and products were collected and a quantitative random risk assessment model (Fig. 2) was established to estimate the probability of releasing ASFV by legal export of pork and pork products from Poland. The model uses @Risk for simulation calculations, using Latin hypercube sampling for 10,000 iterations to generate the probability distribution of each risk node as realistically as possible [27].

The node setting and calculation method for the probability model of ASFV release by exporting pork and pork products are described briefly in Fig. 2 and Table 1.

**Table 1:** The input parameters and calculation method for each node in the model

Notation	Definition	Parameterization	Source	Values
P1	Probability of ASF infection in Poland	P1 = 1-e^(−t × λ)^	[18]	P1 = 0.762
P2	Probability of pig surviving an ASF infection		[28]	Pert (0.05, 0.2, 0.8)
P3	Probability of pigs being selected for slaughter	P3 = Vs/Vp		
Vs	Pig slaughter volume (Thousand head)		[29]	Pert (1344.1, 1765.3, 2465.8)
Vp	Number of fattening pigs (Thousand head)		[29]	Uniform (3856.6, 6490.3)
P4	Survival rate of pigs during transportation	P4 = 1-P4’		
P4’	Mortality of pigs during transportation		[30]	Pert (0.00062, 0.00107, 0.00335)
P5	Probability of infected pigs not being detected in the slaughterhouse		[31]	Beta (1.34, 34.17)
P6	Probability of selecting pork and pork products carrying ASFV to be exported	Beta (α_1_, α_2_)		
α_1_	The quantity of potentially infected pork and pork products in Poland	α_1_ = QI+ 1		
α_2_	The quantity of qualified swine produced in Poland	α_2_ = (QT-QI)-1		
QT	Total production of pork and pork products in Poland		[32]	Triang (969,317, 1,955,500, 2,230,071)
QI	Total amount of pork and pork products with ASFV	QI=NI × Pm × Mp		
NI	Number of infected pigs	NI=Ou × To×Hp		
Ou	Number of ASF undetected outbreaks before official notification		[31]	Pert (1, 1.28, 6)
To	Average herd size in Poland	To = Nt/Sf		
Nt	Pig population in Poland		[33]	Normal (16,189,857, 2,346,461)
Sf	Numbers of pig farms in Poland		[33]	Normal (262,690, 45,068)
Hp	Intra-herd prevalence of ASF		[31]	Pert (0.05, 0.15, 0.32)
Pm	Probability ASF infected pig being transformed into meat	Pm = P2 × P3 × P4 × P5		
Mp	Average weight of each pig in Poland(kg)		[32]	Uniform (85.9, 89.7)
n	Export volume of pork and pork products in Poland (t)		[32]	Triang (9633.8, 9777.9, 645,366)
p	Probability of ASFV carrying pork and pork products exported from Poland	p = P1 × P6		
Pr	Probability of at least 1 t pork and pork Products carrying ASFV exported from Poland	Pr = 1-(1-p) ^n^		

Specifically, the probability of at least 1 t (t) of pork and pork products carrying ASFV exported from Poland through legal channels (Pr) is calculated using formula (2):

2 $$ \Pr =1-{\left(1-\mathrm{p}\right)}^{\mathrm{n}} $$

where n refers to the export volume of pork and pork products in Poland; p (the probability of ASFV carrying pork and pork products exported from Poland) is calculated by the product of 2 conditional probabilities:

3 $$ \mathrm{p}=\mathrm{P}1\times \mathrm{P}6 $$

P1 is the probability of ASF infection in Poland. Due to the ongoing outbreak of ASF in Poland, an exponential function is used to estimate the probability of at least one outbreak within the time interval considered, in the following term:

4 $$ \mathrm{P}1=1-{\mathrm{e}}^{\left(-\mathrm{t}\times \uplambda \right)} $$

where t is the time interval and in this context, t = 1 (representing one month). λ is the average monthly outbreaks of ASF calculated from historical data, and the formula is λ = a/b where a is the total ASF outbreaks in domestic pigs [33] and b is the total number of months during the study period (b = 47, from February 2014 to December 2017).

P6 is the probability of selecting pork and pork products carrying ASFV to be exported. The β distribution is selected to describe this probability, which is described by two parameters α_1_ and α_2_. α_1_ = QI+ 1 represents the quantity of potentially infected pork and pork products in Poland and QI is the total amount of pork and pork products with ASFV. α_2_ = (QT-QI)-1 where QT is the total production of pork and pork products in Poland (FAO) and the parameter α_2_ represents the quantity of qualified pork and pork products in Poland. The distribution of QI can be estimated according to Eq. (5).

5 $$ \mathrm{QI}=\mathrm{NI}\times \mathrm{Pm}\times \mathrm{Mp} $$

where Mp is the average weight of each pig in Poland. Fitting the relevant FAO data to this it is found to be represented by Uniform (85.9, 89.7).

NI indicates the number of infected pigs and is calculated using the formula NI = Ou × To × Hp. In the formula, Ou is the number of ASF undetected outbreaks before official notification and is described by Pert (1, 1.28, 6). To is the average herd size in Poland, To = Nt/Sf where Nt is the pig population in Poland, expressed as Normal (16,189,857, 2,346,461) and Sf is the number of pig farms in Poland, according to the OIE data described as Normal (262,690, 45,068). Hp is the intra-herd prevalence of ASF and this process was described using Pert (0.05, 0.15, 0.32) [31].

The probability of ASF infected pigs being transformed into meat (Pm) is estimated using Eq. (6):

6 $$ \mathrm{P}\mathrm{m}=\mathrm{P}2\times \mathrm{P}3\times \mathrm{P}4\times \mathrm{P}5 $$

P2 is the probability of pigs surviving an ASF infection. According to the literature report [28], Pert (0.05, 0.2, 0.8) distribution may be used to indicate the survival rate.

The pig slaughter volume (Vs) from January 2005 to August 2017 and the average number of fattening pigs per month (Vp) from 2005 to 2016 in Poland were obtained from the European Bureau of Statistics. The formula P3 = Vs/Vp was used to calculate the probability of pigs being selected for slaughter in one month (P3).

P4 was used to indicate the survival rate of pigs during transportation. Due to different transport distances and seasons, there can be differences in mortality of pigs during transportation (P4’). According to the study [30] of neighboring countries (Czech Republic) on the mortality of domestic live pigs during transport, the probability distribution of P4’ node was set to Pert (0.00062, 0.00107, 0.00335). The survival rate of pigs during transportation P4 is equal to 1-P4’.

The probability of infected pigs not being detected in the slaughterhouse is indicated by P5. This process was described using a β-distribution [31] and the parameters were set to Beta (1.34, 34.17).

## Results

### The situation of ASF in Poland

**Fig. 3:**
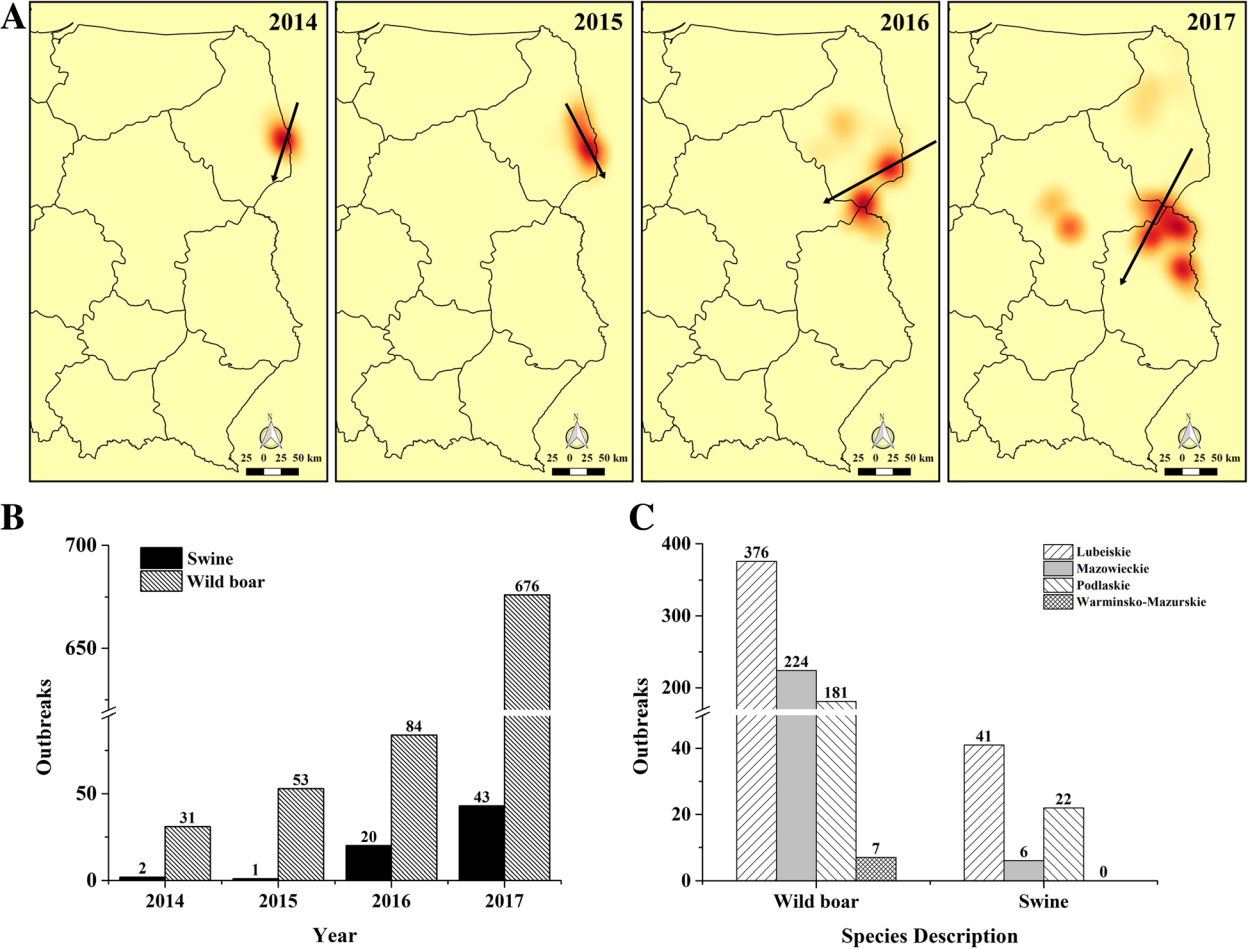
The outbreaks of AFS in Poland during 2014–2017. **a**: The heat map of the outbreak. The darker the area, the more serious the epidemic situation and the arrows represent the disease direction; **b**: The annual outbreak; **c**: The outbreaks in each province. The maps were generated with QGIS Version 2.18 (https://www.qgis.org/en/site/index.html) and the base map was Bing Maps from QuickMapServices

The epidemic in 2014–2015 only broke out in Podlaskie Province (Fig. 3). In 2016, ASF began to spread to neighboring provinces. The main transmission direction of disease is from south to north in Poland from 2014 to 2017. Since 2016, the epidemic has begun to spread to the southwest, and the epidemic area expanded further. By the end of December 2017, infected wild boars were found in 4 provinces (Podlacher, Lublin, Masovian and Warmia-Masuria), and infected domestic pigs were found in 3 provinces (Fig. 3c). The epidemic was most serious in Lublin Province, with a total of 417 outbreaks. The outbreak in the wild boar populations was significantly worse than that in domestic pigs. In 2017, there were 676 wild boar outbreaks in Poland, and this was more than 8 times the outbreak that occurred in 2016 (84). The wild boar epidemic seems to be getting out of control.

### Standard deviation ellipse

**Fig. 4:**
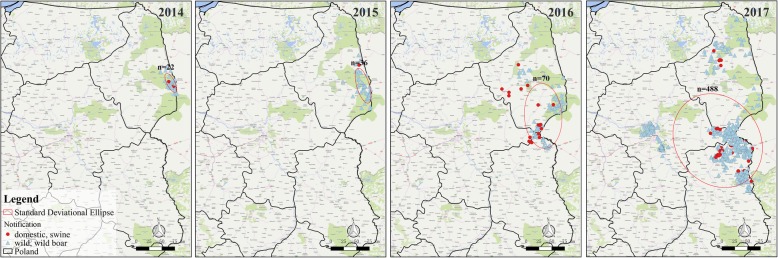
The SDE of the ASF outbreak in Poland in 2014–2017. n: the number of outbreaks in SDE. The maps were generated with QGIS Version 2.18 (https://www.qgis.org/en/site/index.html) and the base map was Bing Maps from QuickMapServices

An epidemic of an infectious disease is closely related to space and time. To analyze the spatial dispersion and directional bias of locations or expansions of the ASF epidemics in Poland Standard Deviation Ellipse (SDE) was used in this work. Figure 4 clearly shows the directional trends of the annual ASF epidemic situation from 2014 to 2017. At the same time, the relevant features of SDE are summarized in Table 2: The longitude coordinates (Center X) and latitude coordinates (Center Y) of the average center, the length of the long axis and the short axis, the ratio of the long axis to the short axis (L/S) and the angle between the long axis and the true north direction (Rotation angle).

**Table 2:** The Parameters of SDE from 2014 to 2017

Year	Center X (°)	Center Y (°)	Long axis(km)	Short axis(km)	L/S^*^	Rotation angle (°)
2014	23.7686	53.1262	63.54	23.93	2.655	133.491
2015	23.7041	53.0793	110.69	38.11	2.905	148.155
2016	23.2904	52.5783	198.85	113.96	1.745	152.177
2017	22.7104	52.1603	301.50	255.66	1.179	99.419

*L/S represent the ratio of Long axis to Short axis

The elliptical center gradually moved southwestwards from 2014 to 2017. The epidemic situation in 2014–2016 has a clear trend, with a general north-south distribution. In 2017, the tendency of the epidemic was reduced (L/S was 1.179) and the distribution was generally northwest-southeast. In 2014 and 2015, the ellipse was confined to the Podlaski Province. Since 2016, the area of the ellipse had significantly expanded and it had begun to cover parts of Lublin and Masovian provinces. In 2017, the area of the ellipse expanded further. This trend is basically consistent with the spread of the epidemic.

### Scan statistics analysis

**Fig. 5:**
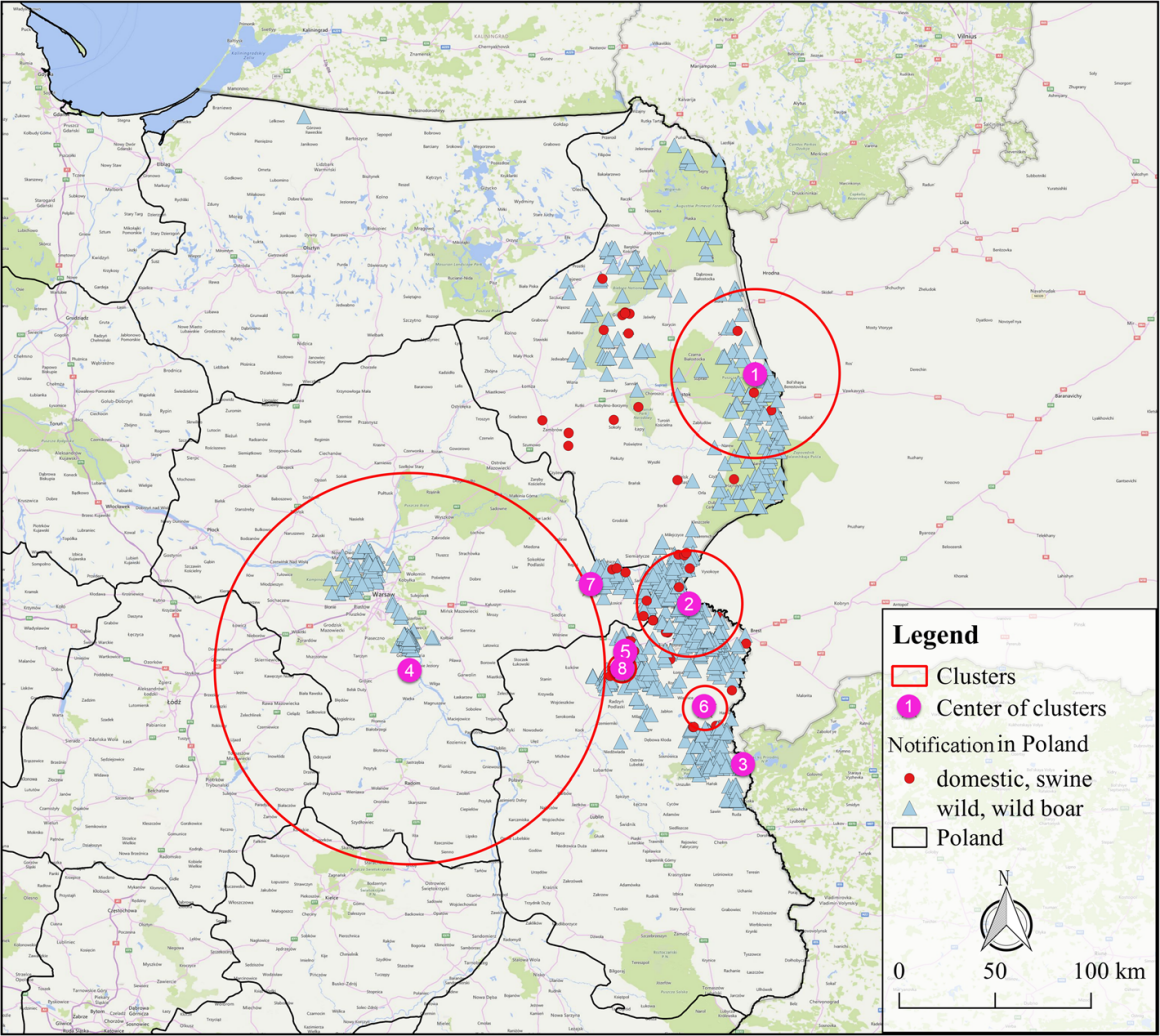
Geographical localization of the ASF clusters in Poland from 2014 to 2017. The maps were generated with QGIS Version 2.18 (https://www.qgis.org/en/site/ index.html) and the base map was Bing Maps from QuickMapServices

In order to make up the fact that SDE measured the dispersion of the epidemic only from the perspective of space, a space–time permutation scan statistic of the ASF epidemic in Poland during 2014–2017 was performed on a daily basis to analyze the clustering situation in both space and time. A total of 8 clusters were obtained (see Table 3 and Fig. 5 for details) and ranked according to the LLR values from highest to the lowest. Among these 8 clusters, 3 of them were mainly involved in wild boar cases. The No. 1 cluster is located on the border area of Podlaskie province near Belarus. It was the earliest area where the ASF broke out in Poland and consequently the infection lasted for a long time. The No. 2 cluster is located at the border between the provinces of Podlaskie, Masovian and Lublin. The number of infected cases was the largest and the duration was the longest in the region. There was a large number of cases of domestic pigs infected. The No. 4 cluster is located near Warsaw, the capital of Poland. The cases in this cluster are all wild boars and the area involved was the largest radius in all the clusters. The onset time of the cluster was shorter but the number of cases was larger. The remaining 5 clusters are with small radii and a little outbreak but a large number of cases were involved with domestic pigs, of which the No. 3 and No. 7 clusters had radii of less than one kilometer. The relative risk values of these clusters were significantly higher than those for wild pigs (such as No. 4 gathering area). The highest RR value of 73.93 was obtained in the No. 7 cluster. Only one domestic pig outbreak was observed over one day, but 19 infected boars were also observed. The relative risks are clearly higher than those surrounding outbreak areas.

**Table 3:** The characteristics of space–time permutation scan statistic results of all the clusters

Cluster ID	No. of outbreaks	Radius (km)	Start	End	Observed cases	Expected cases	RR	LLR
1	145	41.13	2014/2/17	2016/1/9	159	17.03	10.24	219.73
2	228	26.36	2016/8/13	2017/7/14	254	59.16	4.91	187.98
3	2	0	2017/7/24	2017/7/24	46	1.37	34.53	117.78
4	145	98.19	2017/11/24	2017/12/30	206	65.64	3.45	101.78
5	13	4.62	2017/8/17	2017/9/8	48	3.28	15.05	84.77
6	6	11.12	2017/7/27	2017/8/6	26	0.6	44.02	72.76
7	1	0	2017/9/11	2017/9/11	19	0.26	73.93	63.02
8	8	6.96	2017/7/17	2017/7/19	24	0.67	36.35	62.62

### Quantitative risk analysis model

**Fig. 6:**
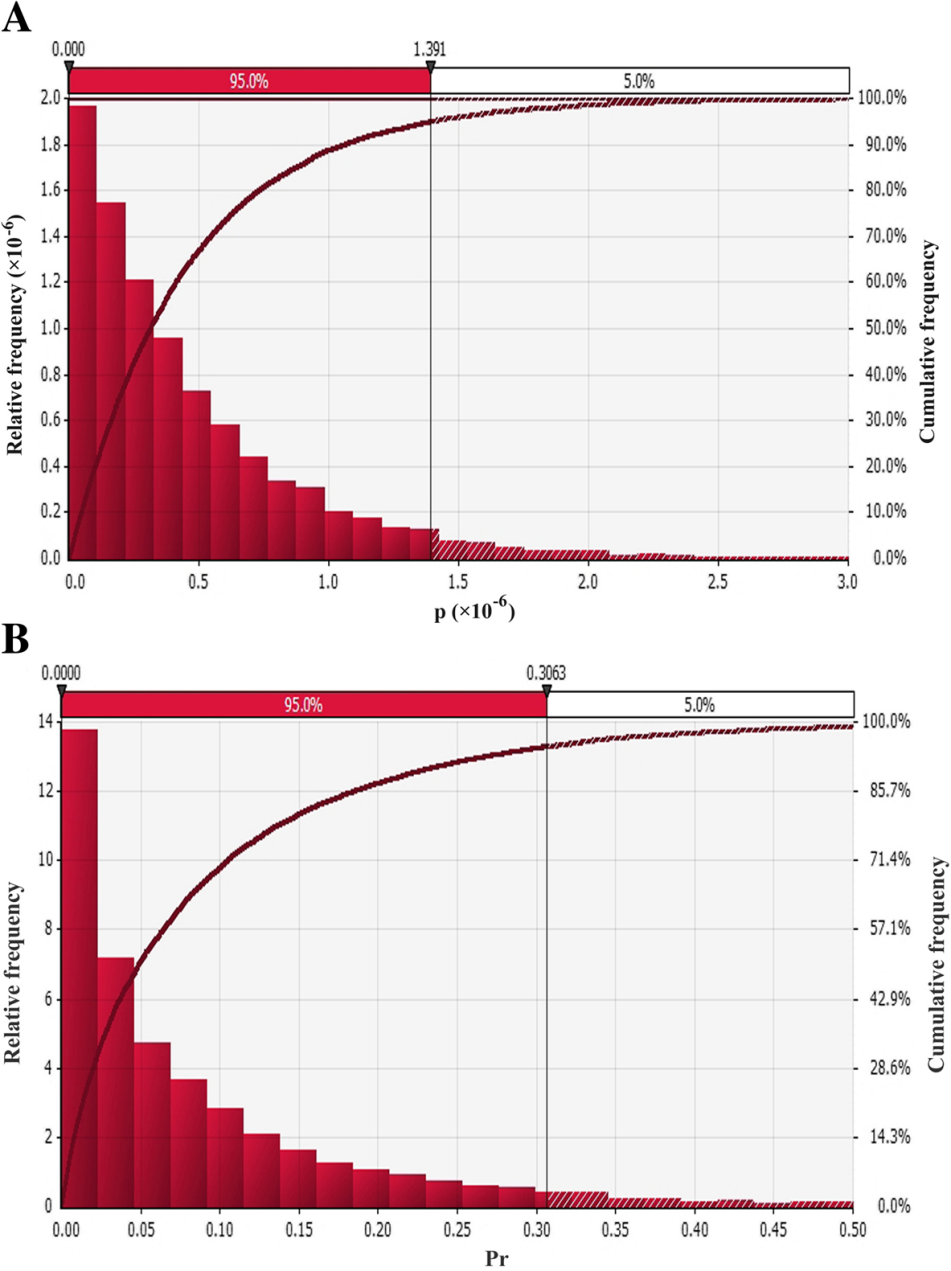
The probability distribution of the output value. **a**: The probability of releasing ASFV by legal export of pork and pork products from Poland (p); **b**: The probability of at least 1 t of pork and pork products carrying ASFV exported from Poland through legal channels (Pr)

The legal export of pork and pork products requires a complicated and rigorous inspection and quarantine process. The main aspects of this process are considered during model construction, to better assess the release route of ASFV. Each node of the model is carried out by 10,000 Latin hypercube sampling, and the output of the model is obtained through calculations. The probability distribution map is shown in Fig. 6. The probability of ASFV carrying pork and pork products exported from Poland (p, shown in Fig. 6a) is 4.577 × 10^− 7^ (the minimum value of this is 1.164× 10^− 11^ and the maximum is 4.384 × 10^− 6^). The probability of at least 1 t of pork and pork products carrying ASFV exported from Poland in one month (Pr) is 0.08 (the minimum and maximum values are 3.961 × 10^− 7^ and 0.922 respectively).

## Discussion

The uncontrolled spread of ASF in Eastern Europe has brought serious risks to pig farming throughout Europe, Central Asia and even China [34]. In February 2014, Poland officially notified the authorities of the first ASF infected wild boar in its territory. Since then, the ASF began to spread in Poland (Fig. 3). As of December 2017, the epidemic continued and there was a trend of further spreading. This paper mainly analyzes the directional trend and the clustering situation of ASF in Poland. SDE is a common method for exploring whether the transmission of an infectious disease shows directional trends [35–37]. Our results show the directional trend of ASF in Poland from 2014 to 2016 is changing from a general north-south to northwest-southeast (Fig. 4). We speculate that the transmission of the disease is affected mainly by the epidemic occurring in wild boar population. The results of De la Torre et al. [38] agree with our conjecture that the reasons for the introduction of ASF in the Baltic States and Poland are related to the wild boar habitat suitability and the neighboring distance from infected wild boars and domestic pigs.

Time and spatial distribution play important roles in the description and analysis of diseases [39, 40]. The space-time permutation scan statistics were used for all ASF outbreak data from 2014 to 2017 in Poland and eight clusters were obtained (Fig. 5). Space-time permutation scan statistics is used to explore the clustering of infectious diseases. As no presupposition is made on the size, location and scale of clustering, the selection bias is avoided, the data information can be mined to the maximum, and the existence of aggregation can therefore be found [24]. The analysis by Grzegorz Woźniakowski et al. [7] showed that the affected wild boar was able to migrate from 0.5 to 25 km, but the most frequent distance was 1–10 km in 2014–2015. In our study The radius of the largest cluster (the No. 4 cluster, and all the cases in this cluster are wild boar) is 98.19 km. I. Iglesias Martin et al. [41] also used the permutation model to identify regions and periods of time in which notifications of ASF in wild boar occurred in the EU from 2 years (2014–2015). Nine significant (*P* < 0.005) time–space clusters of ASF cases were detected, which were located in Lithuania, Poland, Latvia and Estonia and the most clusters occurred between September and December. Our results show that the outbreak time of domestic pigs occurred mainly from July to September. The clusters involving wild boars have too a long time to gather and it had a wide range of influence, but the outbreak trends all increased from October in 2014–2017. The results were basically same to the study by Cappai et al. [42] of ASF persistence in Sardinia, and the results were consistent with the peak of domestic pigs’ notifications in May and early summer, while the wild boar peak from October to February. It can be seen that in the summer when the domestic pig epidemic was concentrated in very few areas, pig farmers were able to further strengthen the implementation of prevention and control measures.

The main purpose of the import risk analysis is to provide importing countries with an objective and reliable method of assessing the risks associated with imported animal and animal related products (i.e. genetic material, feedstuffs, biological products and pathological materials) [43]. Therefore, risk analysis should always be transparent and based on the available information and full reference, as their results can be used to regulate international trade [31]. L. Mur et al. [44] have constructed a quantitative model to assess the risk of introducing ASFV into the EU through the legal import of live pigs. The model results show that the annual introduction probability of ASFV in the whole EU is 5.22 × 10^–3^, and the risk of introducing ASFV into Poland is the highest. They also assessed the risk of introducing ASFV into the European Union by constructing a semi-quantitative model based on a weighted combination of risk factors to evaluate the three transportation routes (returning trucks and wastes from international ships and planes) [45]. The results show that the relative risk of introducing ASFV to most EU countries through transport related routes is relatively low, but Poland and Lithuania have higher risk levels. The returning trucks is the riskiest route for ASFV to be introduced into the EU.

Finally, it is necessary to point out some limitations of the model in this study. The legal export needs to undergo a strict inspection and quarantine. China has been importing pork and pork products from Poland for a short time. Since the onset of outbreak of ASF in Poland in February 2014, China has terminated the trade in pigs, pork and its products between China and Poland. We are unable to obtain enough related data to be used for modeling, so this article only evaluates the probability of releasing ASFV by legal export of pork and pork products from Poland. The main links of pork and its products from slaughtering to export are taken into account in the article. The prediction of the model is based on the worst-case scenario: that the infected pork and pork products are not detected in all surveillance tests and are therefore eligible for export. And our model also does not account for wild boar as a means for transmission of virus between domestic pig enterprises. We acknowledge that wild boars are likely to have an important impact on disease spread [46], but it is so complicated and controversy associated with the estimation of this pathways that the collection of the detailed data was out of the scope and possibilities of this study. But once enough data is available, we should also analyze other possible export routes, such as export of other biological products (such as semen and embryos), animal feed and other illegal routes, including aircraft and other internationally transported wastes.

## Conclusions

This paper uses GIS to analyze the distribution, the trend of epidemic situation and the clustering of ASF in Poland. In order to limit the spread of the epidemic, more effective prevention and control measures should be taken in the highly prevalent areas of the epidemic season (for the domestic pigs this is in the summer and for wild boars this is mainly in winter). Subsequently, the collected data can be used to construct a quantitative risk assessment, to further analysis of the probability of releasing ASFV by legal export of pork and pork products from Poland through legal channels. This type of assessment helps to assist decision makers and helps countries to trade scientifically with Polish pork and pork products. Risk analysis is a dynamic and on-going process as new data become available the model can be improved in order to consider new and evolving situations.

## Abbreviations

ASF: African swine fever
ASFV: African swine fever virus
Center X: Longitude coordinates of the average center
Center Y: Latitude coordinates of the average center
EMPRES-i: EMPRES Global Animal Disease Information System
EU: European Union
FAO: United Nations Food and Agriculture Organization
GIS: Geographic Information System
Hp: Intra-herd prevalence of ASF
L/S: Ratio of the long axis to the short axis
LLR: Log likelihood ratio statistics
Mp: Average weight of each pig in Poland
n: Export volume of pork and pork products in Poland
NI: Number of infected pigs
Nt: Pig population in Poland
OIE: World Organisation for Animal Health
Ou: Number of ASF undetected outbreaks before official notification
p: Probability of ASFV carrying pork and pork products exported from Poland
P1: Probability of ASF infection in Poland
P2: Probability of pig surviving an ASF infection
P3: Probability of pigs being selected for slaughter
P4: Survival rate of pigs during transportation
P4’: Mortality of pigs during transportation
P5: Probability of infected pigs not being detected in the slaughterhouse
P6: Probability of selecting pork and pork products carrying ASFV to be exported
Pm: Probability ASF infected pig being transformed into meat
Pr: Probability of at least 1 t pork and pork Products carrying ASFV exported from Poland
QI: Total amount of pork and pork products with ASFV
QT: Total production of pork and pork products in Poland
RR: Relative risk value
SDE: Standard Deviation Ellipse
Sf: Numbers of pig farm in Poland
To: Average herd size in Poland
Vp: Number of fattening pigs
Vs: Pig slaughter volume
α1: The quantity of potentially infected pork and pork products in Poland
α2: The quantity of qualified swine produced in Poland
